# Supplementary material for: Feed Clusters According to In Situ and In Vitro Ruminal Crude Protein Degradation
Source: Animals (Basel). 2023 Jan 7;13(2):224. doi: 10.3390/ani13020224 (PMC9855172; doi:10.3390/ani13020224)
Supplement: Supplementary file 1 [file animals-13-00224-s001.zip › animals-2127448-supplementary.pdf]

Supplementary material

Table S1
Estimated microbial nitrogen in the residues of *in situ* crude protein degradation at the various incubation times

Feedstuff	2 h	4 h	6 h	8 h	16 h	24 h	48 h	72 h
Barley	4.6	8.6	12.1	15.1	23.6	28.4	33.4	n.a.
Wheat	3.9	7.3	10.3	12.8	20.1	24.1	28.4	29.2
Wheat bran	4.1	7.6	10.7	13.3	20.8	25.0	29.4	30.2
DDGS	3.4	6.4	9.1	11.3	17.9	21.7	26.0	26.8
CGF	4.3	8.0	11.2	14.0	21.8	26.2	30.9	31.7
Soybeans	2.3	4.3	6.1	7.6	12.0	14.5	17.4	17.9
SBM	1.3	2.4	3.4	4.3	6.8	8.2	9.8	10.1
SFM	3.5	6.6	9.2	11.6	18.3	22.2	26.5	
RSMa et	3.0	5.6	7.9	9.9	15.6	18.9	22.7	23.4
RSMb ot	3.2	6.0	8.5	10.6	16.8	20.4	24.4	25.1
RSMc n	2.2	4.1	5.8	7.3	11.5	14.0	16.7	17.3
RSM et	2.2	4.2	5.9	7.3	11.6	14.1	16.8	17.3
RSM n	2.3	4.3	6.1	7.6	12.0	14.6	17.4	18.0
RSM ft	2.3	4.4	6.2	7.7	12.2	14.8	17.7	18.3
Lupin *Boregine* n	0.7	1.3	1.8	2.3	3.5	4.2	5.0	5.1
Lupin *Boregine* t	3.2	6.0	8.4	10.6	16.7	20.3	24.2	25.0
Lupin *Boruta* n	3.2	6.0	8.4	10.6	16.7	20.3	24.2	25.0
Lupin *Boruta* t	3.2	5.9	8.3	10.4	16.5	20.0	23.9	24.6
Pea *Hardy*	1.6	3.0	4.3	5.3	8.3	10.0	11.8	n.a.
Pea *Astronaute*	1.4	2.6	3.7	4.6	7.2	8.6	10.2	n.a.
Pea *Navarro*	0.9	1.7	2.4	3.0	4.7	5.6	6.6	n.a.
SBP	6.2	11.5	16.2	20.2	31.5	37.9	44.6	45.8
GS I	11.9	21.3	28.8	34.7	48.3	53.6	56.8	57.0
GS I*	11.6	20.8	28.1	33.9	47.2	52.4	55.6	55.8
GS II	11.9	21.3	28.8	34.7	48.3	53.6	56.8	57.0
GS II*	11.8	21.1	28.4	34.3	47.7	53.0	56.2	56.4
GS III	12.2	21.8	29.4	35.4	49.3	54.8	58.1	58.3
GS III*	12.1	21.7	29.3	35.3	49.1	54.6	57.9	58.1
GS IV	11.9	21.3	28.8	34.7	48.3	53.6	56.8	57.0
GS IV*	11.8	21.2	28.6	34.4	47.9	53.2	56.4	56.6
GS V	12.0	21.5	29.1	35.0	48.8	54.2	57.4	57.6
GS V*	11.9	21.3	28.8	34.7	48.3	53.6	56.8	57.0
GS VI	11.9	21.3	28.8	34.7	48.3	53.6	56.8	57.0
GS VI*	11.9	21.3	28.8	34.7	48.3	53.6	56.8	57.0
GS VII	12.2	21.9	29.5	35.5	49.5	55.0	58.3	58.5
GS VII*	11.9	21.4	28.9	34.8	48.4	53.8	57.0	57.2
GS VIII	12.0	21.5	29.0	34.9	48.6	54.0	57.2	57.4
GS VIII*	12.1	21.6	29.2	35.2	49.0	54.4	57.6	57.8

* Grass silage ensiled with bacterial inoculant; CGF: corn gluten feed; CP: crude protein; DDGS: dried distillers’ grains with solubles; GS: grass silage; RSM: rapeseed meal; SBM: soybean meal; SBP: sugar beet pulp; SFM: sunflower meal.
All estimates are given as % of total nitrogen.
The estimations were performed as described by Parand and Spek (2021).

Table S2

Estimated parameters of ruminal *in situ* crude protein degradation at 72 h and 24 h incubation time considering the effect of the correction for microbial nitrogen contained in the degradation residues [a]

Feedstuff	Uncorrected degradation (72 h)							Corrected degradation (72 h)							Uncorrected degradation (24 h)							Corrected degradation (24 h)						
	a	*b*	*c*	*L*	ED$_2$	ED$_5$	ED$_8$	*a*	*b*	*c*	*L*	ED$_2$	ED$_5$	ED$_8$	*a*	*b*	*c*	*L*	ED$_2$	ED$_5$	ED$_8$	*a*	*b*	*c*	*L*	ED$_2$	ED$_5$	ED$_8$
Barley	35	58	34	0.00	90	86	82	35	59	38	0.00	91	87	83	35	57	37	0.00	89	85	81	35	59	38	0.00	91	87	83
Wheat	52	48	22	3.34	91	82	76	53	47	23	2.99	92	84	78	54	46	23	4.06	91	82	76	53	47	23	2.99	92	84	78
Wheat bran	48	46	31	0.94	90	85	81	48	47	33	0.89	91	87	83	48	52	33	1.01	89	85	81	48	47	33	0.89	91	87	83
DDGS	57	38	10	0.00	88	82	78	54	35	20	0.00	86	82	79	54	32	19	0.00	83	80	77	54	35	20	0.00	86	82	79
CGF	61	33	26	0.00	92	89	87	61	33	33	0.00	92	89	87	64	29	33	0.67	90	88	86	61	33	33	0.00	92	89	87
Soybeans	55	45	10	1.07	91	83	77	57	42	12	2.25	92	84	79	56	43	10	1.70	91	83	77	57	42	12	2.25	92	84	79
SBM	15	85	14	3.56	84	67	55	17	83	15	3.91	84	68	56	17	81	19	4.53	83	67	55	17	83	15	3.91	84	68	56
SFM	20	76	19	0.17	88	79	72	20	76	21	0.16	89	81	74	20	75	20	0.21	88	79	72	20	76	21	0.16	89	81	74
RSMa et	13	78	13	0.00	80	69	61	12	78	15	0.00	81	71	63	12	77	14	0.00	79	68	61	12	78	15	0.00	81	71	63
RSMb ot	10	77	7	0.10	70	55	45	10	72	11	0.25	70	58	50	10	67	9	0.41	65	53	45	10	72	11	0.25	70	58	50
RSMc n	23	64	305	2.63	80	71	65	19	79	10	0	85	72	63	19	80	9	0.03	84	69	60	19	79	10	0	85	72	63
RSMc et	43	51	12	0.00	87	79	74	43	51	16	0	88	81	76	45	44	165	1.30	84	78	74	43	51	16	0	88	81	76
RSMd	24	69	21	1.69	85	75	68	25	68	24	1.70	86	77	70	24	67	23	1.80	84	75	68	25	68	24	1.70	86	77	70
RSMd ft	12	88	4	3.91	66	44	34	11	58	12	1.99	57	45	38	11	66	8	5.68	55	39	30	12	48	161	2.64	51	42	36
Lupin *Boregine* n	32	67	25	1.75	91	82	75	28	72	22	0.98	92	82	75	27	72	22	0.99	92	82	75	28	72	22	0.98	92	82	75
Lupin *Boregine* t	33	64	161	3.42	87	77	69	32	68	15	2.12	89	77	69	32	68	14	2.41	88	76	67	32	68	15	2.12	89	77	69
Lupin *Boruta* n	32	67	21	1.03	91	82	75	32	67	21	0.96	92	83	77	32	67	20	1.01	92	82	75	32	67	21	0.96	92	83	77
Lupin *Boruta* t	29	71	12	2.67	86	72	62	25	75	12	1.03	87	74	65	26	74	11	1.92	86	72	62	25	75	12	1.03	87	74	65
Pea *Hardy*	47	52	21	2.26	92	84	78	45	55	20	1.40	93	85	79	45	55	19	1.50	93	84	78	45	55	20	1.40	93	85	79
Pea *Astronuate*	44	55	154	1.53	92	84	79	42	58	14	0.25	92	84	77	47	52	166	2.40	92	84	78	42	58	14	0.25	92	84	77
Pea *Navarro*	48	52	18	3.30	92	82	76	42	58	14	0.88	92	83	76	44	56	15	1.78	92	82	76	42	58	14	0.88	92	83	76
SBP	21	75	11	0.00	84	72	64	22	77	13	0.00	89	77	69	21	79	10	0.00	87	73	65	22	77	13	0.00	89	77	69
GS I	74	19	13	0.66	90	87	85	74	22	16	0.31	94	91	89	74	18	15	1.37	89	86	84	74	22	16	0.31	94	91	89
GS I*	73	20	11	0.50	90	86	84	73	24	14	0.00	94	91	88	73	20	11	0.98	90	86	84	73	24	14	0.00	94	91	88
GS II	73	19	13	1.39	89	85	83	72	24	16	0.35	93	90	88	73	18	16	1.76	88	85	83	72	24	16	0.35	93	90	88

GS II*	70	22	15	2.61	88	84	81	69	28	14	0.30	93	89	86	70	21	18	2.76	87	84	81	69	28	14	0.30	93	89	86
GS III	72	19	14	2.67	88	84	82	72	25	15	0.63	93	89	87	72	19	14	2.20	87	84	82	72	25	15	0.63	93	89	87
GS III*	71	20	14	1.47	88	84	82	71	25	17	0.84	93	89	87	71	19	17	2.20	87	84	82	71	25	17	0.84	93	89	87
GS IV	73	19	13	2.77	88	84	82	73	24	14	0.78	93	89	87	73	18	14	2.88	87	84	82	73	24	14	0.78	93	89	87
GS IV*	72	19	12	2.45	87	84	81	71	24	15	0.78	92	89	86	71	18	14	2.36	87	83	81	71	24	15	0.78	92	89	86
GS V	69	21	14	2.75	87	83	80	69	27	16	0.98	92	88	86	69	20	17	2.87	86	83	80	69	27	16	0.98	92	88	86
GS V*	68	23	13	1.79	87	83	80	68	28	17	0.78	92	88	86	68	20	165	2.18	86	83	81	68	28	17	0.78	92	88	86
GS VI	69	22	11	1.43	87	83	81	70	26	15	0.67	93	89	86	70	19	15	2.65	86	83	81	70	26	15	0.67	93	89	86
GS VI*	71	21	12	2.99	87	83	80	70	27	12	0.34	93	89	86	70	20	14	3.02	87	83	80	70	27	12	0.34	93	89	86
GS VII	65	26	14	2.58	86	81	78	64	32	15	0.66	92	87	84	66	23	18	3.20	85	81	78	64	32	15	0.66	92	87	84
GS VII*	67	24	13	2.48	87	82	79	66	30	15	0.48	92	88	85	67	23	14	2.37	86	82	79	66	30	15	0.48	92	88	85
GS VIII	65	26	12	2.35	87	82	78	64	32	14	0.32	92	87	84	65	25	15	2.58	86	81	78	64	32	14	0.32	92	87	84
GS VIII*	64	27	13	2.37	86	81	77	63	34	14	0.42	92	87	83	64	27	154	2.89	86	81	78	63	34	14	0.42	92	87	83
Range of SD	0.00-6.78	0.11-10.63	0.33-254.99	0.00-3.62	0.24-4.69	0.53-5.45	0.28-6.15	0.00-7.75	0.00-29.47	0.54-20.21	0.00-3.78	0.07-16.38	0.31-9.22	0.16-5.80	0.00-7.67	0.50-30.00	0.56-260.02	0.00-7.67	0.25-16.79	0.37-7.92	0.38-5.52	0.00-7.75	0.00-26.82	0.54-257.62	0.00-3.78	0.07-15.07	0.31-8.05	0.16-4.62

* Grass silage ensiled with bacterial inoculant; *a*: washout protein, which is instantly disappearing; *b*: potentially degradable protein; *c*: rate of crude protein degradation; CGF: corn gluten feed; DDGS: dried distillers' grains with solubles; GS: grass silage; *L*: lag time; RSM: rapeseed meal; SBM: soybean meal; SBP: sugar beet pulp. SD: standard deviation; SFM: sunflower meal.

a and *b* are given as %; *c* is given as % h^{-1}; *L* is given as h.

Color markings show significant differences with kruskal wallis test between ED of all variants ($p < 0.05$).

[a] According to Parand and Spek (2021)

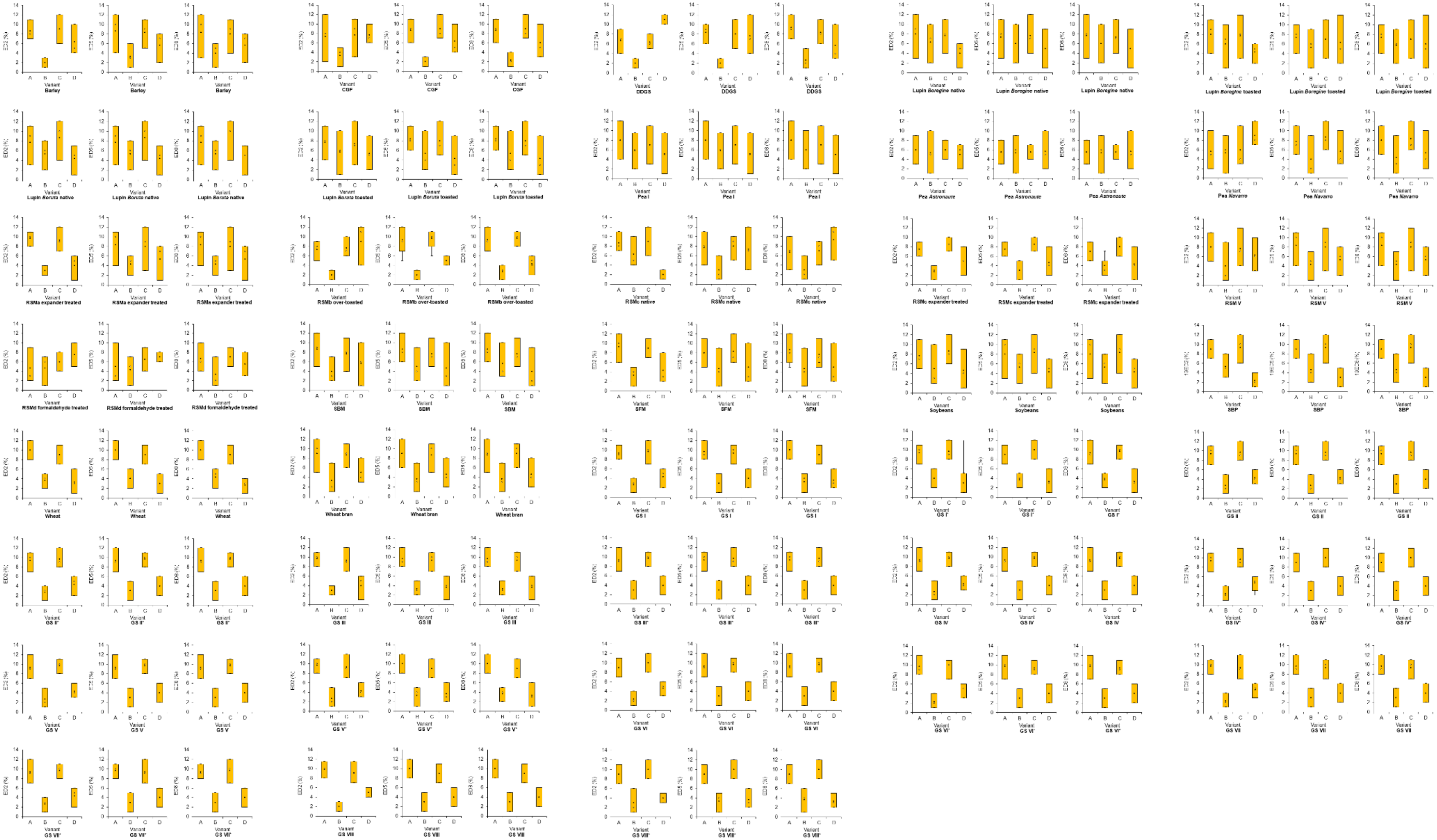
Barley
CGF
DDGS
Lupin Boregine native
Lupin Boregine toasted
Lupin Boruta native
Lupin Boruta toasted
Pea I
Pea Astronaute
Pea Navarro
RSMa expander treated
RSMb over-toasted
RSMc native
RSMc expander treated
RSM V
RSMd formaldehyde treated
SBM
SFM
Soybeans
SBP
Wheat
Wheat bran
GS I
GS I'
GS II
GS II'
GS III
GS III'
GS IV
GS IV'
GS V
GS V'
GS VI
GS VI'
GS VII
GS VII'
GS VIII
GS VIII'
Variant
ED2 (%)
ED5 (%)
ED8 (%)

* Grass silage ensiled with bacterial inoculant; CGF: corn gluten feed; DDGS: dried distillers' grains with solubles; GS: grass silage; RSM: rapeseed meal; SBM: soybean meal; SBP: sugar beet pulp SFM: sunflower meal.
Variant A: ED corrected for microbial nitrogen at 24 h incubation time; variant B: ED uncorrected for microbial nitrogen at 24 h incubation time; variant C: ED corrected for microbial nitrogen at 72 h incubation time; variant D: ED uncorrected for microbial nitrogen at 72 h incubation time.

Figure S1

Effect of the correction for microbial nitrogen contained in the degradation residues and reduction of maximal incubation time on *in situ* effective crude protein degradation (ED) at 0.02 h^{-1} (ED_2), 0.05 h^{-1} (ED_5) and 0.08 h^{-1} (ED_8) assumed ruminal passage rate

Table S3

Estimated parameters of ruminal *in vitro* crude protein degradation at 24 h incubation time

Feedstuff	*a*	*b*	*c*	*L*
Barley	31	14	20	0.07
Wheat	39	36	31	0.67
Corn	22	4	18	0
Wheat bran	34	26	27	0
DDGS	22	38	15	0
CGF	42	11	9	0
Soybeans	18	56	26	0
SBM	23	61	30	0
SFM	46	38	25	0.01
RSMa et	22	52	15	0.05
RSMb ot	21	43	13	0.08
RSMc n	18	66	9	0.33
RSMc et	14	66	14	0.10
RSMd n	25	55	12	0
RSMd ft	10	90	3	0.54
Faba bean	66	-1	13	0
Lupin *Boregine* n	74	15	29	0.08
Lupin *Boregine* t	36	43	29	0
Lupin *Boruta* n	70	18	31	0
Lupin *Boruta* t	37	42	38	0
Pea *Hardy*	73	8	15	0.01
Pea *Astronaute*	74	8	28	1.08
Pea *Navarro*	75	8	13	0.47
Sugar beet pulp	30	24	7	0.69
GS I	73	6	9	0.86
GS I*	68	11	14	0.23
GS II	71	10	11	0.11
GS II*	67	12	8	0.28
GS III	72	8	16	0.73
GS III*	69	11	7	0
GS IV	72	9	11	0.59
GS IV*	71	11	8	0.56
GS V	70	10	9	0.23
GS V*	68	11	9	0.22
GS VI	70	11	10	0.62
GS VI*	70	12	7	0.66
GS VII	66	13	9	0.08
GS VII*	67	14	8	0.18
GS VIII	65	14	11	0.17
GS VIII*	64	16	9	0.26
Range of SD	0.10-13.02	0.35-11.52	0.06-14.90	0.00-1.71

* Grass silage ensiled with bacterial inoculant; *a*: washout protein; *b*: potentially degradable protein; *c*: rate of crude protein degradation; CGF: corn gluten feed; DDGS: dried distillers' grain with solubles; *L*: lag time; RSM: rapeseed meal; SBM: soybean meal; SD: standard deviation; SFM: sunflower meal. *a* and *b* are given as %; *c* is given as % h^{-1}; *L* is given as h.

Table S4

Comparison of *in situ* and *in vitro* estimates of effective crude protein degradation (ED) at 0.02 h^{-1} (ED_2), 0.05 h^{-1} (ED_5) and 0.08 h^{-1} (ED_8) assumed ruminal passage rate and 24 h incubation time

	ED_2		ED_5		ED_8	
Feedstuff	*In situ*	*In vitro*	*In situ*	*In vitro*	*In situ*	*In vitro*
Barley	91^{aA}	43^{bB}	87^{aA}	42^{bB}	83^{aA}	40^{bB}
Wheat	92^{aA}	73^{bB}	84^{aA}	70^{bB}	78^{aA}	67^{bB}
Wheat bran	91^{aA}	58^{bA}	87^{aA}	56^{bA}	83^{aA}	54^{bA}
DDGS	86^{aA}	55^{bB}	82^{aA}	50^{bB}	79^{aA}	46^{bB}
CGF	92^{aA}	51^{bA}	89^{aA}	49^{bA}	87^{aA}	48^{bA}
Soybeans	92^{aA}	70^{bB}	84^{aA}	65^{bB}	79^{aA}	61^{bB}
SBM	84^{aA}	80^{bB}	68^{aA}	75^{bB}	56^{aA}	71^{bB}
SFM	89^{aA}	81^{bB}	81^{aA}	77^{bB}	74^{aA}	75^{aA}
RSMa et	81^{aA}	67^{bB}	71^{aA}	60^{bB}	63^{aA}	55^{bB}
RSMb ot	70^{aA}	58^{bB}	58^{aA}	52^{bB}	50^{A}	47^{B}
RSMc n	85^{aA}	71^{bA}	72^{aA}	59^{bA}	63^{aA}	52^{bA}
RSMc et	88^{A}	72^{A}	81^{A}	62^{A}	76^{aA}	55^{bA}
RSMd n	86^{aA}	72^{bA}	77^{aA}	64^{bA}	70^{aA}	58^{bA}
RSMd ft	51^{aA}	62^{aA}	42^{aA}	41^{aA}	36^{A}	32^{A}
Lupin *Boregine* n	92^{aA}	88^{bB}	82^{aA}	87^{bB}	75^{aA}	86^{bB}
Lupin *Boregine* t	89^{aA}	76^{bB}	77^{aA}	73^{bA}	69^{aA}	70^{aA}
Lupin *Boruta* n	92^{aA}	87^{bB}	83^{aA}	86^{aA}	77^{aA}	84^{bB}
Lupin *Boruta* t	87^{aA}	77^{bA}	74^{aA}	74^{aA}	65^{aA}	72^{bB}
Pea *Hardy*	93^{aA}	80^{bB}	85^{aA}	79^{bB}	79^{aA}	78^{aA}
Pea *Astronaute*	92^{aA}	81^{bA}	84^{aA}	81^{aA}	77^{aA}	80^{aA}
Pea *Navarro*	92^{A}	82^{A}	83^{aA}	81^{aA}	76^{aA}	80^{bA}
Sugar beet pulp	89^{aA}	47^{bA}	77^{aA}	42^{bA}	69^{aA}	39^{bA}
GS I	94^{aA}	78^{bA}	91^{aA}	77^{bA}	89^{aA}	76^{bA}
GS I*	94^{aA}	77^{bA}	91^{aA}	75^{bA}	88^{aA}	74^{bA}
GS II	93^{aA}	79^{bA}	90^{aA}	77^{bA}	88^{aA}	76^{bA}
GS II*	93^{aA}	77^{bA}	89^{aA}	75^{bA}	86^{aA}	74^{bA}
GS III	93^{aA}	78^{bA}	89^{aA}	76^{bA}	87^{aA}	75^{bA}
GS III*	93^{aA}	78^{bA}	89^{aA}	76^{bA}	87^{aA}	74^{bA}
GS IV	93^{aA}	79^{bA}	89^{aA}	78^{bA}	87^{aA}	77^{bA}
GS IV*	92^{aA}	79^{bA}	89^{aA}	77^{bA}	86^{aA}	76^{bA}
GS V	92^{aA}	78^{bA}	88^{aA}	76^{bA}	86^{aA}	75^{bA}
GS V*	92^{A}	77^{A}	88^{aA}	75^{bA}	86^{aA}	74^{bA}
GS VI	93^{aA}	79^{bA}	89^{aA}	77^{bA}	86^{aA}	76^{bA}
GS VI*	93^{aA}	79^{bA}	89^{aA}	77^{bA}	86^{aA}	75^{bA}
GS VII	92^{aA}	77^{bA}	87^{aA}	75^{bA}	84^{aA}	73^{bA}
GS VII*	92^{aA}	78^{bA}	88^{aA}	75^{bA}	85^{aA}	73^{bA}
GS VIII	92^{aA}	77^{bA}	87^{aA}	75^{bA}	84^{A}	73^{A}
GS VIII*	92^{aA}	77^{bA}	87^{aA}	74^{bA}	83^{aA}	72^{bA}
Range of SD	0.07-15.08	0.29-3.68	0.31-8.05	0.24-3.16	0.16-4.62	0.17-3.70

* Grass silage ensiled with bacterial inoculant; ^{ab} different lower-case superscripts mark significant differences with *t*-test between *in situ* and *in vitro* ED ($p < 0.05$); ^{AB} different upper-case superscripts mark significant differences with Wilcoxon rank sum test between *in situ* and *in vitro* ED ($p < 0.05$); CGF: corn gluten feed; CP: crude protein; DDGS: dried distillers' grain with solubles; GS: grass silage; RSM: rapeseed meal; SBM: soybean meal; SD: standard deviation; SFM: sunflower meal.

Effective crude protein degradation is given in %/CP.

The *in situ* crude protein degradation data were corrected for the amount of microbial nitrogen present in the feed residues at each specific incubation time using the equations of Parand and Spek (2021).

Table S5

Parameter describing clusters

degQ2					degQ5					degQ8					degQ2, degQ5, degQ8				
No. of Clusters	Clusters		R^2	Min. Dist.	No. of Clusters	Clusters		R^2	Min. Dist.	No. of Clusters	Clusters		R^2	Min. Dist.	No. of Clusters	Clusters		R^2	Min. Dist.
37	Lupin *Boregine* t	Pea Hardy	1.00	0	37	RSMc n	RSMd n	1.00	0	37	Lupin *Boregine* t	SFM	1.00	0	37	GS I	GS V*	1.00	0
36	Lupin *Boruta* t	Pea *Navarro*	1.00	0	36	Pea *Astronaute*	SFM	1.00	0	36	RSMa et	Wheat	1.00	0	36	GS V	GS VI*	1.00	0
35	RSMa	RSMb ot	1.00	0	35	RSMc et	Soybean	1.00	0	35	RSMd ft	GS I	1.00	0	35	GS III	GS VII*	1.00	0
34	RSMc n	RSMd n	1.00	0	34	RSMa et	GS I	1.00	0	34	CL36	GS II	1.00	0	34	RSMa et	GS VIII	1.00	0
33	Lupin *Boruta* n	SBM	1.00	0	33	CL37	GS I*	1.00	0	33	CL34	GS III	1.00	0	33	CL37	GS VIII*	1.00	0
32	CL35	GS I	1.00	0	32	Wheat	GS II*	1.00	0	32	CL35	GS III*	1.00	0	32	RSMc n	RSMd n	1.00	0.04
31	RSMc et	GS I*	1.00	0	31	CL34	GS III	1.00	0	31	GS IV*	GS V	1.00	0	31	GS IV*	GS VI	1.00	0.04
30	CL32	GS II*	1.00	0	30	CL31	GS III*	1.00	0	30	CL32	GS V*	1.00	0	30	CL35	GS VII	1.00	0.04
29	CL34	GS III	1.00	0	29	GS IV	GS IV*	1.00	0	29	CL31	GS VI	1.00	0	29	GS IV	CL31	1.00	0.04
28	CL29	GSIII*	1.00	0	28	GS II	GS V	1.00	0	28	CL29	GS VI*	1.00	0	28	CL34	CL33	1.00	0.04
27	GS II	GS IV	1.00	0	27	CL30	GS V*	1.00	0	27	CL33	GS VII	1.00	0	27	CL28	CL30	1.00	0.04
26	CL37	GS IV*	1.00	0	26	CL29	GS VI	1.00	0	26	CL27	GS VII*	1.00	0	26	CL27	GS II	1.00	0.04
25	CL27	GS V	1.00	0	25	CL28	GS VI*	1.00	0	25	CL26	GS VIII	1.00	0	25	CL26	GS III*	1.00	0.04
24	CL30	GS V*	1.00	0	24	CL25	GS VII	1.00	0	24	CL30	GS VIII*	1.00	0	24	CL25	CL36	0.99	0.04
23	CL25	GS VI	1.00	0	23	CL27	GS VII*	1.00	0	23	RSMc n	RSMd n	1.00	0.06	23	CL24	CL29	0.99	0.04
22	CL23	GS VI*	1.00	0	22	CL23	GS VIII	1.00	0	22	CL24	GS II*	1.00	0.06	22	CL23	GS II*	0.99	0.06
21	CL28	GS VII	1.00	0	21	CL22	GS VIII*	1.00	0	21	GS IV	CL28	1.00	0.06	21	CL22	GS I*	0.99	0.07
20	CL21	GS VII*	1.00	0	20	CL21	CL24	1.00	0.07	20	Lupin *Boruta* n	Lupin *Boruta* t	1.00	0.06	20	CL21	CL32	0.99	0.09
19	CL24	GS VIII	1.00	0	19	Pea *Hardy*	RSMd ft	1.00	0.07	19	CL25	CL22	0.99	0.06	19	Lupin *Boregine* t	Pea *Hardy*	0.99	0.09

18	CL19	GS VIII*	1.00	0	18	Lupin *Boregine* t	CL19	0.99	0.07	18	CL23	GS I*	0.99	0.06	18	Pea *Astronaute*	Pea *Navarro*	0.99	0.12
17	CL18	CL31	1.00	0.09	17	CGF	SBP	0.99	0.07	17	CL19	CL18	0.99	0.06	17	CL19	CL18	0.99	0.13
16	CL26	CL22	0.99	0.09	16	CL20	CL32	0.99	0.07	16	CL17	CL21	0.99	0.06	16	CL17	SFM	0.99	0.14
15	CL36	Pea *Astronaute*	0.99	0.09	15	CL16	CL33	0.99	0.07	15	CL37	Pea *Astronaute*	0.99	0.12	15	CGF	SBP	0.99	0.15
14	Lupin *Boregine* n	CL33	0.999	0.09	14	CL15	CL26	0.99	0.07	14	CL15	Pea *Hardy*	0.99	0.12	14	CL20	Wheat	0.99	0.16
13	CL17	CL20	0.99	0.09	13	CL18	CL36	0.99	0.14	13	CL14	Pea *Navarro*	0.99	0.12	13	Lupin *Boregine* n	Lupin *Boruta* n	0.99	0.20
12	CL16	CL13	0.99	0.09	12	Lupin *Boruta* t	Pea *Navarro*	0.99	0.14	12	CGF	SBP	0.99	0.12	12	CL16	Lupin *Boruta* t	0.98	0.25
11	DDGS	Wheat bran	0.99	0.09	11	CL14	RSMb ot	0.99	0.14	11	CL12	DDGS	0.99	0.12	11	CL12	RSMb ot	0.98	0.25
10	CL15	SFM	0.99	0.18	10	CL13	CL12	0.99	0.14	10	Lupin *Boregine* n	CL20	0.99	0.18	10	CL11	CL14	0.89	0.26
9	CL12	CL10	0.97	0.18	9	Lupin *Boregine* n	Lupin *Boruta* n	0.99	0.22	9	CL13	RSMb ot	0.98	0.23	9	CL13	CL10	0.81	0.26
8	CGF	SBP	0.97	0.27	8	CL9	CL10	0.97	0.22	8	RSMc et	Soybean	0.98	0.23	8	DDGS	Wheat bran	0.81	0.26
7	CL9	Wheat	0.96	0.27	7	CL8	CL11	0.83	0.22	7	CL10	CL9	0.96	0.29	7	RSMc et	Soybean	0.81	0.28
6	CL7	Soybean	0.95	0.27	6	DDGS	Wheat bran	0.82	0.22	6	CL16	CL8	0.93	0.3	6	CL15	CL8	0.81	0.42
5	CL14	CL6	0.89	0.36	5	CL7	SBM	0.75	0.36	5	Barley	CL11	0.93	0.35	5	CL9	CL7	0.77	0.43
4	Barley	CL8	0.88	0.45	4	Barley	CL17	0.75	0.43	4	CL7	CL6	0.69	0.35	4	Barley	CL6	0.75	0.44
3	CL4	CL11	0.85	0.63	3	CL4	CL6	0.73	0.43	3	CL5	Wheat bran	0.68	0.35	3	CL5	SBM	0.66	0.54

2	CL3	CL5	0.15	10.73	2	CL5	CL35	0.69	0.43	2	CL3	CL4	0.17	0.47	2	CL4	CL3	0.04	0.86
1	CL2	RSMd ft	0	1.16	1	CL3	CL2	0	0.94	1	CL2	SBM	0	0.76	1	CL2	RSMd ft	0	0.92

*: GS ensiled with bacterial inoculant; Cl: cluster; DDGS: dried distillers' grains with solubles; Dist.: distance; et: expander-treated ft: formaldehyde-treated; GS: grass silage; Min.: minimum; n: native; No.: number ot: over-toasted; R^2: coefficient of determination; RSM: rapeseed meal; SBM: soybean meal; SFM: sunflower meal; t: treated.

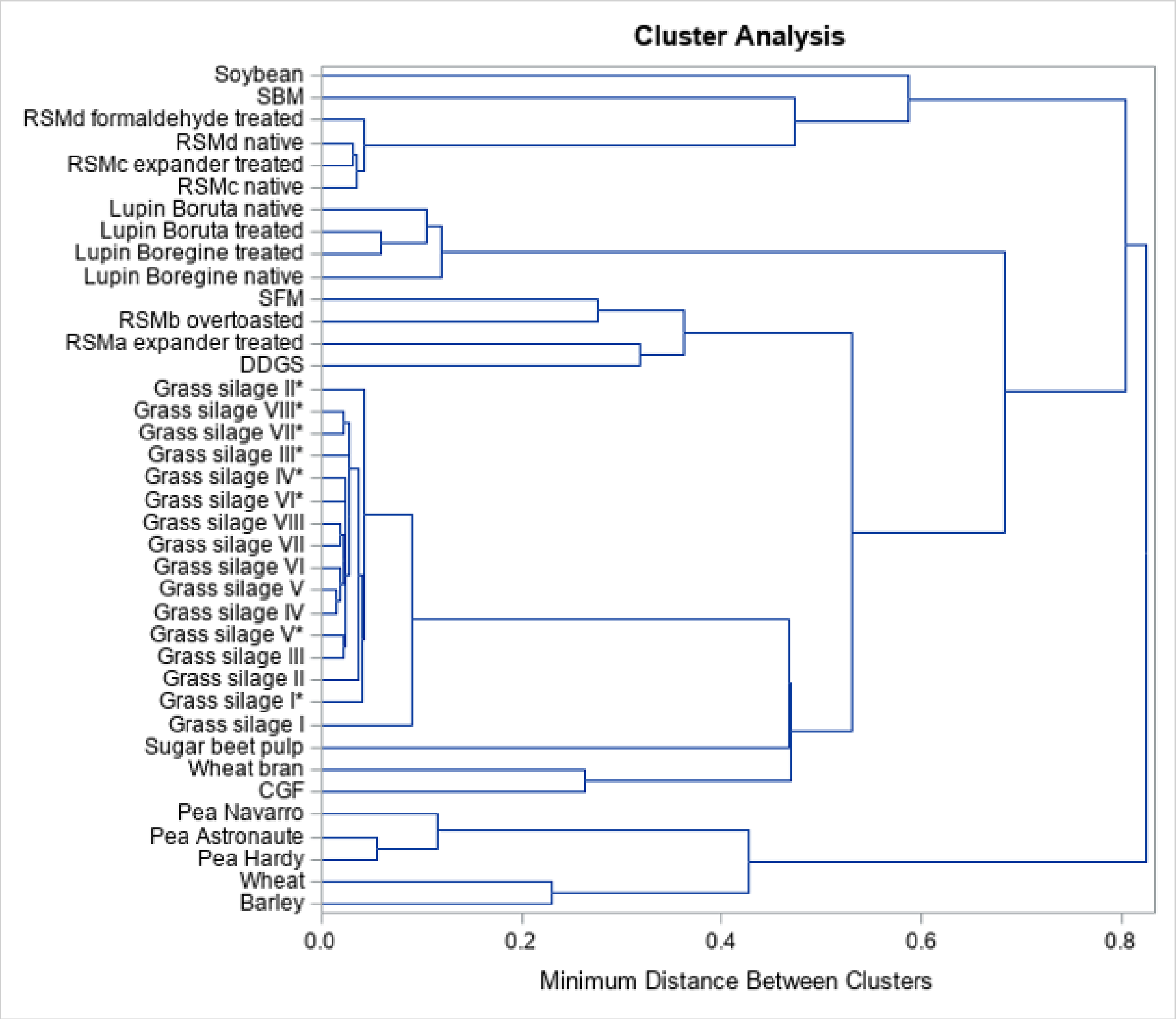

*: GS ensiled with bacterial inoculant; CGF: corn gluten feed; DDGS: dried distillers' grains with solubles; GS: grass silage; RSM: rapeseed meal; SBM: soybean meal; SBP: sugar beet pulp; SFM: sunflower meal.

Figure S2

Cluster analysis including crude nutrients, detergent fibers and starch of used feedstuffs

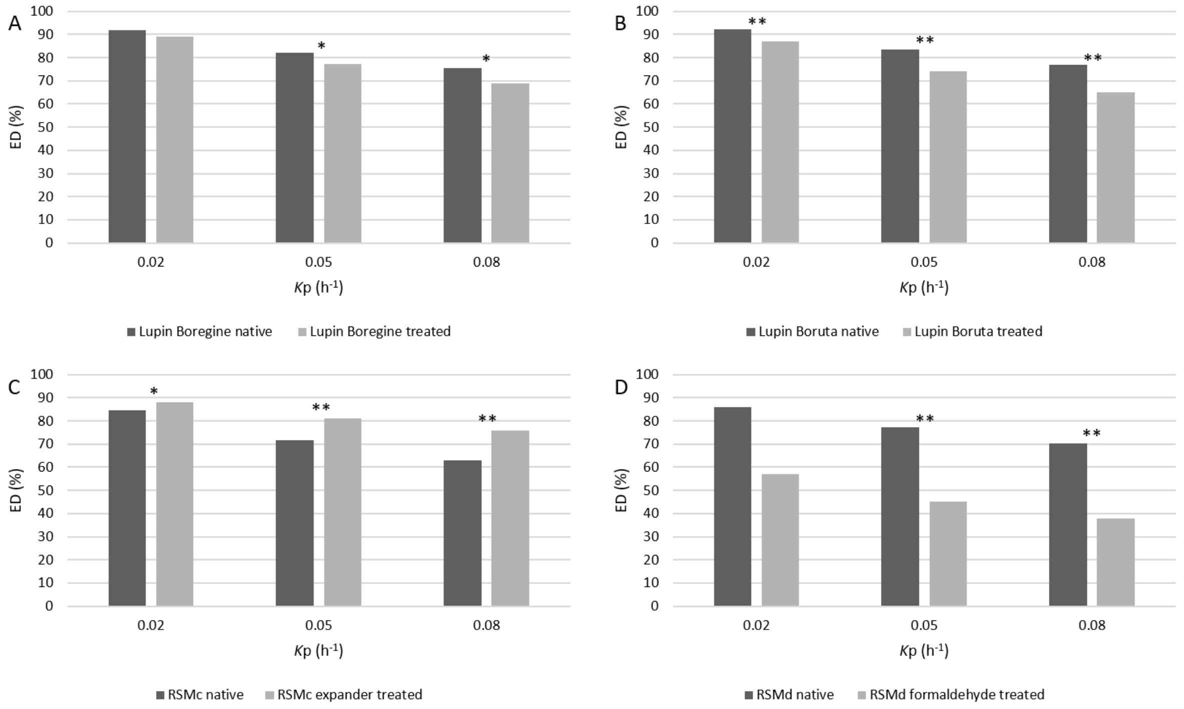

Asterisks indicate significant differences between native and treated materials: * $p < 0.05$, ** $p < 0.01$, *** $p < 0.001$; RSM: rapeseed meal.

Figure S3

Effective crude protein degradation (ED) at 0.02 h^{-1}, 0.05 h^{-1} and 0.08 h^{-1} ruminal passage rate estimated *in situ* in native and treated feedstuff: lupin *Boregine* (A), lupin *Boruta* (B), RSMc (C) and RSMd (D) (72 h incubation time).
